# Predictors of COVID-19 Mortality in Hemodialysis Patients, Hamadan Province, Iran

**DOI:** 10.34172/jrhs.2022.77

**Published:** 2022-02-03

**Authors:** Ebrahim Jalili, Salman Khazaei, Ali Reza Soltanian, Seyed Mehdi Hosseini, Saeid Bashirian, Samareh Ghelichkhani, Toos Kiani, Somayeh Akbari, Leila Halimi

**Affiliations:** ^1^Associate Professor of Emergency Medicine, Department of Emergency Medicine, School of Medicine, Besat Hospital, Hamadan University of Medical Sciences, Hamadan, Iran; ^2^Department of Epidemiology, School of Public Health, Hamadan University of Medical Sciences, Hamadan, Iran; ^3^Research Center for Health Sciences, Hamadan University of Medical Sciences, Hamadan, Iran; ^4^Department of Biostatistics, School of Public Health, Hamadan University of Medical Sciences, Hamadan, Iran; ^5^Modeling of Noncommunicable Diseases Research Center, Hamadan University of Medical Sciences, Hamadan, Iran; ^6^Department of Internal Medicine, School of Medicine, Hamadan University of Medical Sciences, Hamadan, Iran; ^7^Department of Public Health, School of Public Health, Hamadan University of Medical Sciences Hamadan, Iran; ^8^Social Determinants of Health Research Center, Health Sciences and Technology Research Institute, Hamadan University of Medical Sciences, Hamadan, Iran; ^9^Deputy of Treatment, Hamadan University of Medical Sciences, Hamadan, Iran; ^10^Shahid Beheshti Hospital, Hamadan University of Medical Sciences, Hamadan, Iran; ^11^Researcher at Clinical Research Development Unit of Shahid Beheshti Hospital, Hamadan University of Medical Sciences, Hamadan, Iran

**Keywords:** COVID-19, Hemodialysis patients, Mortality, Renal failure

## Abstract

**Background:** Identification of the predictors of coronavirus disease 2019 (COVID-19)-related death in hemodialysis patients plays a key role in the management of these patients. In this regard, the present study aimed to evaluate the predictors of death among COVID-19 infected hemodialysis patients in Hamadan province, Iran.

**Study design:** A cross-sectional study.

**Methods:** This cross-sectional study investigated 50 COVID-19 infected hemodialysis patients who were confirmed by polymerase chain reaction (PCR) test and referred to hemodialysis wards of hospitals located in Hamadan province, Iran, from March 2019 and January 2020. In order to compare the demographic characteristics and clinical variables between survived and deceased patients, the independent student *t* test and chi-square test were applied.

**Results:** Out of 50 confirmed COVID-19 hemodialysis patients, 27 (54%) cases were male, 38 (76%) subjects were urban residents, and 4 (8%) individuals were smokers. A significant relationship was observed between patients’ gender, age, acute respiratory distress syndrome (ARDS) status, and body mass index (BMI) with the treatment outcome (*P*<0.05). A significantly higher level of serum albumin was observed in the survived patients (3.49±0.37 vs. 3.17±0.42, *P* = 0.030). Moreover, in terms of lactate dehydrogenase (LDH) level, a significantly higher level of LDH was observed in the patients who died (1471.1±1484.89 vs. 670.86±268.85, *P* = 0.005).

**Conclusions:** It can be concluded that some demographic characteristics of the patients, including age, gender, ARDS status, BMI, co-morbidities, and laboratory signs and symptoms are associated with disease outcomes in COVID-19 infected hemodialysis patients. Therefore, awareness about the predictors of death in these patients can help make better and direct clinical decisions and inform health officials about the risk of COVID-19 mortality among hemodialysis patients.

## Background

 Coronavirus disease 2019 (COVID-19) as the first coronavirus pandemics has attracted worldwide attention called Public Health Emergency of International Concern.^1^ On January 30, 2020, the World Health Organization (WHO) declared that the outbreak of novel coronavirus-2019 pandemics is a Public Health Emergency of International Concern.^2^ Currently, the novel coronavirus disease is prevalent in most countries over the world, mainly among patients with co-morbidities. Patients had a fever, and some others showed shortness of breath with chest radiographs suggesting the symptoms of coronavirus.^3^ At the onset of the disease, common symptoms include fever, cough, and bruising, while other signs are the production of sputum, headache, bleeding, diarrhea, indigestion, and the occurrence of lymphopenia.^4^

 The findings of the epidemiological studies indicated that the COVID-19-related mortality rate was higher among patients affected by a history of chronic diseases, including diabetes, hypertension, and chronic renal failure.^5^ Chronic kidney disease (CKD) is a global main health challenge^6^ and is defined according to a stage with the renal function below 50% of their normal ability.^7^ If the kidneys fail to function more than 10%-15% of their normal ability, the final stage of kidney disease (end-stage renal disease [ESRD]) occurs. At this stage, kidney transplantation, hemodialysis, or peritoneal dialysis is essential for patient survival.^6^

 According to Iranian official reports, the annual increase of ESRD prevalence is 11%, and some evidence shows that the number of these patients will be doubled for the next five years.^8^ In Iran, ESRD prevalence and incidence has raised from 137 (13.8 incidences per million population) in 1997 to 238 (49.9 incidences per million population) in 2000 and 357 (63.8 incidences per million population) in 2006.^9^ Almost 54% of Iranian ESRD patients receive hemodialysis care and the others need kidney transplantation.^10^

 The results of clinical studies showed that the risk of COVID-19 infection is more than 10% and 6.4% among hospitalized hemodialysis patients and healthcare staff, respectively.^11^ On the other hand, many COVID-19 patients are exposed to high risks for acute renal failure. This condition causes a high number of hospital admissions and frequent entry to the intensive care unit (ICU) and is recognized as responsible for many pulmonary, cardiovascular, and renal complications and in the final stage of progressive and irreversible destruction of renal function. In a study, Ma et al. found that inflammatory cytokines were lower in hemodialysis patients with COVID-19 than in other patients.^12^ Another important factor is related to the decreased serum activity of the angiotensin-converting enzyme (ACE2) being prevalent in hemodialysis patients.^13^

 Generally, these patients are exposed to a high risk for the COVID-19 occurrence according to the weak immune system, high rate of co-morbidity for other chronic diseases (e.g., diabetes and hypertension), and frequent referral for hospital hemodialysis care.^14^ It is essential to better understand those death-associated factors among hemodialysis patients with COVID-19 since a designed study has not been conducted in Iran yet, and the range of clinical disease burden includes widely from asymptomatic carriers to patients affected by severe final stages. Therefore, the identification of the associated factors of increased mortality risk among hemodialysis patients with COVID-19 can help healthcare professionals in order to triage these patients, better conduct treatment management, and recognize those patients in need of intensive care.

## Methods

 This descriptive-analytical research evaluated the predictors of COVID-19-related mortality among hemodialysis patients in Hamadan province, Iran. A total of 50 COVID-19 confirmed cases were diagnosed among hemodialysis patients in Hamadan province during 10 months (from March 2019 to January 2020). Peritoneal hemodialysis cases and patients with acute renal failure were excluded from the study, and only ESRD cases were evaluated. For collecting the required data, the researcher reviewed patient records including demographic characteristics (e.g., gender, age, marital status, education level, and occupational status), clinical data (e.g., clinical symptoms, time of referral, real test results, related diseases, CT scan results, radiographic results, and dialysis-related factors [i.e., vascular access, dialysis duration per week, dialysis sessions per week, and duration of disease diagnosis]), and laboratory findings (e.g., temperature, saturation of peripheral oxygen, pulse rate, white blood cell, lymphocyte, hemoglobin, platelet, albumin, blood urea nitrogen, creatinine, lactate dehydrogenase (LDH), prothrombin time, international normalized ratio, erythrocyte sedimentation rate, systolic and diastolic blood pressure (BP), and the final discharge status [death or recovery]). In this study, the mortality predictors among hemodialysis patients with COVID-19 were studied. Quantitative variables were reported as mean ± SD, and frequency (%) was used to express categorical variables. An independent t-test was also employed to compare quantitative variables between two or more groups. Chi-square/Fisher’s exact tests were utilized to compare qualitative variables in two groups. Data analysis was performed using Stata 14.0 software, and a *P* value less than 0.05 was considered statistically significant.

## Results

**Figure 1:**
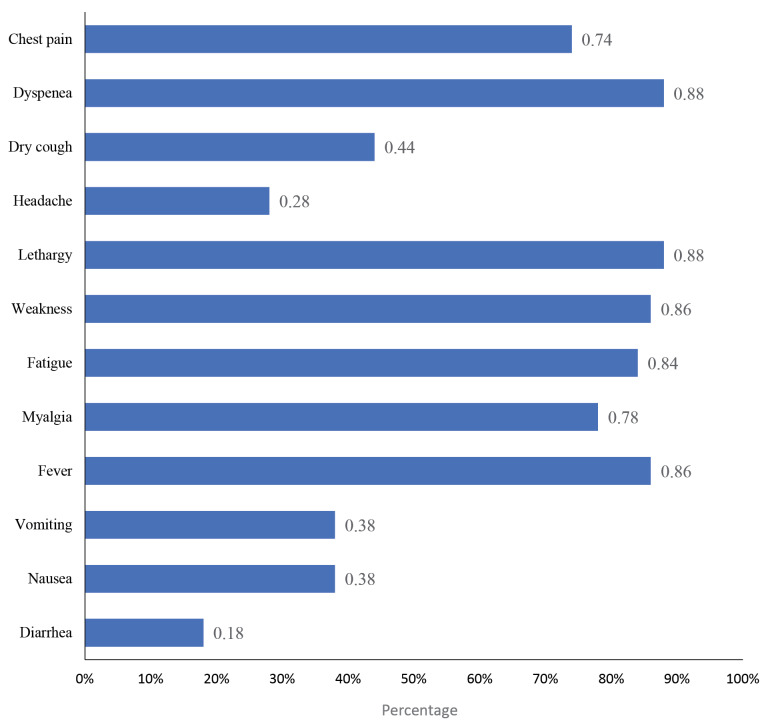
Common signs and symptoms of the hemodialysis patients with COVID-19.

 The present study investigated 50 COVID-19 infected hemodialysis patients who were confirmed by polymerase chain reaction (PCR) test and referred to the hemodialysis wards of hospitals in Hamadan, Iran, from March 2019 to January 2020. As shown in Figure 1, dyspnea, lethargy, weakness, and fever are observed among 88%, 88%, 86%, and 86% of the studied patients, respectively, which can be considered the most common signs and symptoms among COVID-19 patients.

Table 1 presents the demographic characteristics of the investigated patients, 27 (54%) cases of whom were male and 38 (76%) subjects were urban residents. Moreover, 28 (56%) patients had normal body mass index (BMI) and 4 (8%) cases were smoker. Comorbidity with diabetes, hypertension, and cardiovascular disease among patients were 62%, 78%, and 28%, respectively. In this study, 15 (30%) patients died. Gender, age, acute respiratory distress syndrome (ARDS) status, and BMI of the patients showed a significant association with treatment outcome (*P* < 0.05).

**Table 1:** Characteristics of the hemodialysis patients with COVID-19 according to their treatment outcome

**Variables**	**Total**		**Remission**		**Death**		***P *** **value**
	**Number**	**Percent**	**Number**	**Percent**	**Number**	**Percent**	
Gender							0.016
Female	23	46.0	20	87.0	3	13.0	
Male	27	54.0	15	56.0	12	44.0	
Place of residency							0.770
Urban	38	76.0	27	71.0	11	29.0	
Rural	12	24.0	8	67.0	4	33.0	
Age group (year)							0.050
<50	16	32.0	14	87.0	2	13.0	
50-65	16	32.0	12	75.0	4	25.0	
>65	18	36.0	9	50.0	9	50.0	
Smoking							0.040
No	46	92.0	34	74.0	12	26.1	
Yes	4	8.0	1	25.0	3	75.0	
Body mass index (kg/m^2^)							0.044
18.5-25	28	56.0	23	82.0	5	18.0	
25-29.9	16	36.0	10	63.0	6	38.0	
≥ 30	6	12.0	2	33.0	4	67.0	
Diabetes							0.660
Yes	31	62.0	21	60.0	10	67.0	
No	19	38.0	14	40.0	5	33.0	
Hypertension							0.330
Yes	39	78.0	26	74.0	13	87.0	
No	11	22.0	9	26.0	2	13.0	
Cardiovascular disease							0.220
Yes	14	28.0	8	23.0	6	40.0	
No	36	72.0	27	77.0	9	60.0	
Acute respiratory distress syndrome							0.001
Yes	9	18.0	2	22.0	7	78.0	
No	41	82.0	33	80.0	8	20.0	
Anticoagulant use							0.300
Yes	32	36.0	24	75.0	8	25.0	
No	18	64.0	11	61.0	7	39.0	
Vascular access							0.090
Catheter	19	38.0	9	7.0	10	53.0	
Fistula	18	36.0	12	67.0	6	33.0	
Graft	7	14.0	7	100	0	0	
Sheldon	6	12.0	3	50.0	3	50.0	
ICU hospitalization							0.005
Yes	7	14.0	1	14.0	6	86.0	
No	43	86.0	30	67.0	13	30.0	

Table 2 summarizes the comparison of the clinical and biochemical variables according to the treatment outcome of the patients. Survived patients had significantly higher serum albumin levels, compared to the deceased cases (3.49 ± 0.37 vs. 3.17 ± 0.42, *P* = 0.030). Furthermore, the amount of LDH was significantly higher in the deceased cases, compared to survived patients (1471.1 ± 1484.89 vs. 670.86 ± 268.85, *P* = 0.005). Moreover, these patients had higher systolic BP (134.14 ± 21.86 vs. 120.26 ± 18.41, *P* = 0.030).

**Table 2:** Comparison of the clinical and biochemical variables among COVID-19 hemodialysis patients with remission and death outcome

**Variables**	**Remission**		**Death**		***P*** **value**
	**Mean**	**SD**	**Mean**	**SD**	
Temperature	37.20	0.4	37.25	0.6	0.720
Saturation of peripheral oxygen	88.48	5.7	88.50	7.8	0.990
Pulse Rate	90.42	16.0	89.07	16.5	0.790
Weekly dialysis time	10.44	2.3	10.07	3.1	0.650
White blood cell	8097.14	30.5	8800	5005.2	0.550
Lymphocyte	17.34	9.4	16.69	11.1	0.840
Hemoglobin	10.01	1.9	9.80	1.5	0.710
Platelet	178.49	82.1	162.71	72.3	0.530
Albumin	3.49	0.4	3.17	0.4	0.030
Blood urea nitrogen	104.51	48.0	97.64	35.4	0.630
Creatinine	10.32	10.5	6.93	3.0	0.250
Lactate dehydrogenase	670.86	268.9	1471.10	1484.9	0.005
Prothrombin time	13.24	8.0	17.67	7.1	0.110
International normalized ratio	1.73	2.4	1.63	1.0	0.900
Partial thromboplastin time	29.48	11.6	31.59	11.2	0.590
Erythrocyte sedimentation rate	70.51	29.3	64.56	30.1	0.600
Systolic blood pressure	120.26	18.4	134.14	21.9	0.030
Diastolic blood pressure	71.18	12.7	73.07	16.4	0.670

## Discussion

 This cross-sectional study evaluated the predictors of COVID-19-related mortality among hemodialysis patients. The important findings of the present study indicated that the demographic characteristics of the patients, including age, gender, BMI, ARDS condition, place of residency, underlying diseases, as well as laboratory signs and symptoms (e.g., low albumin levels and elevated LDH) were associated with renal disease outcomes in hemodialysis patients with COVID-19. In a similar study, there was an apparent difference in COVID-19-related mortality rates and the raised mortality risk in elderly patients receiving renal replacement therapy and male hemodialysis patients.^15^ These results are consistent with the reported data related to the median age of 67 years old to be affected with COVID-19 among hemodialysis patients; however, they show a contradiction with the findings of the present study regarding women with a higher incidence of COVID-19.^16^

 According to the results of a study by Kikuchi et al, the higher mortality rate in hemodialysis patients with COVID-19 may be related to age. The majority of COVID-19 cases were aged 70-90 years old in hemodialysis patients; however, in the general population, they ranged between 20 and 60 years old ^17^. In the present study, from 50 hemodialysis patients with COVID-19, 76% of the cases lived in urban areas that was consistent with data obtained from a study by Hsu et al based on admission rates in urban dialysis centers with a significant relationship between hemodialysis clients in urban clinics and the incidence rate of COVID-19. This finding may result from the raised chance for the development of COVID-19 among people settled in more congested areas.^18^

 According to the results, a significant relationship was observed between ARDS condition and the death rate of hemodialysis patients with COVID-19. Cytokine release storm can be attributed to a proposed mechanism for ARDS condition and COVID-19-related multi-organ dysfunction that leads to a regular release of proinflammatory cytokines, followed by ACE2-mediated viral attacks to host alveolar cells, such as interleukins, especially interleukin 6 (IL-6), and tumor necrosis factor-alpha. It has been suggested that the immune system through a modulator effect in the uremia may decrease partially the proinflammatory cytokines in the hemodialysis patients. The hemodialysis patients are generally prone to the COVID-19 infection due to the suppressed immune system, though they generally experience mild symptoms during the infection, compared to healthy individuals. On the other hand, the suppressed immune system can increase the mortality rate among this group of patients.^19^

 However, according to the previous studies, patients who already had kidney diseases were probably found with a remarkably higher sensitivity to COVID-19-related complications.^20^ Therefore, the COVID-19 associated consequences can be more severe in patients with kidney dysfunction, compared to COVID-19 patients with healthy renal conditions.^21^ These symptom differences can be explained partially with innate immune deficiency based on some functional defects of neutrophils, monocytes, as well as B and T cells, vascular dysfunction, and increased inflammation that are common in the advanced CKD.^22^ According to the findings, the common signs and symptoms were reported as shortness of breath (88%), lethargy (88%), weakness (86%), and fever (86%) among patients in this study in descending order. Furthermore, the rates of underlying diabetes, hypertension, and cardiovascular disease were 62%, 78%, and 28% among the study samples, respectively. In a Wuhan-based study with 627 hemodialysis patients at a hemodialysis center, diabetic patients were found to be associated with a higher infection risk.^9^

 Moreover, according to the previous studies, those patients with underlying kidney failure have reported respiratory symptoms (shortness of breath and coughing) and fever, compared to COVID-19 patients without CKD, suggesting the required attention to COVID-19 warning signs.^20^ Based on the results obtained, the high serum level of albumin is attributed to high survival rates. Moreover, the raised level of LDH is highly associated with mortality rates in the subjects. Previous studies have also demonstrated a statistical association between hypoalbuminemia and ischemic heart disease, as well as an independent higher risk of occurrence of deaths in hemodialysis patients.^23^ It has been estimated that during the first 28 days of COVID-19 and hospitalization, the mortality rates are about 25% and 33.5%, respectively.

 The Spanish Society of Nephrology has reported a mortality rate of 24.9% among 600 hemodialysis patients in less than three weeks. In the mentioned study, of 50 hemodialysis patients with COVID-19, 15 (30%) cases passed away.^2,15^ These findings are in agreement with the results of the present study. COVID-19 epidemics had an obvious influence on the mortality rate of all COVID-19 patients with renal disorders. It is noteworthy that these symptoms are more severe in elderly renal patients and showed a higher rate among kidney transplant recipients.^20^ Due to the important role of hemodialysis centers in the transmission of infection, it is necessary to provide a critical protocol for infection and crisis control in future pandemics to increase patient safety and decrease adverse health effects.^15^ The mortality data and predictors can be useful for guiding clinical decisions and informing public and health authorities about the risk of COVID-19 mortality among both kidney transplant and hemodialysis patients. Therefore, it is necessary to pay special attention to the warning signs of COVID-19 in this group.^24^

 Comprehensive follow-up of the COVID-19 hemodialysis patients during admission in the hospital to discharge, death, or 28 days after infection is considered the strength of this study. However, some limitations can be addressed in the present study, such as a limited number of cases, which decreased the power of the study and limited information regarding the severity of the disease. Moreover, the treatment option as potential predictors of treatment outcome was not evaluated.

## Conclusion

 This study indicated some demographic characteristics of COVID-19 infected hemodialysis patients including age, gender, ARDS status, BMI, co-morbidities, and laboratory signs (e.g., low albumin levels and increased LDH) that were attributed to the disease outcome. Therefore, awareness about the predictors of death in these patients can help make direct clinical decisions and inform public and health officials about the risk of COVID-19 mortality among hemodialysis patients. Therefore, the awareness of these predictors can be useful in reducing complications and death, as well as accelerate the identification of treatment options, care, and preventive measures for these patients.

## Acknowledgments

 The authors thank the Vice Chancellor for Research and Technology of Hamadan University of Medical Sciences, Iran for approving this study and the Vice Chancellor for Treatment and Hospitals of Hamadan University of Medical Sciences, Iran for their cooperation in gathering the information required for this project.

## Conflict of interests

 The authors declare that they have no conflict of interest.

## Funding

 The study was funded by the Hamadan University of Medical Sciences, Hamadan, Iran (No. 9911148147).

**Highlights**

- Higher levels of serum albumin are associated with higher survival rates in COVID-19 patients.
- The patients’ gender, age, ARDS status, and BMI are associated with the treatment outcome of COVID-19 patients.
- The amount of LDH is associated with higher survival of COVID-19 patients.
